# The role of the multidisciplinary tumor board after endoscopic resection of malignant tumors: is it worth it?

**DOI:** 10.1007/s00464-023-10555-3

**Published:** 2023-11-22

**Authors:** Konstantinos Kouladouros, Maximilian Centner, Christoph Reissfelder, Sebastian Belle, Georg Kähler

**Affiliations:** 1 Central Interdisciplinary Endoscopy Department, University Medical Center Mannheim, Medical Faculty Mannheim, University of Heidelberg, Theodor-Kutzer-Ufer 1-3, 68167 Mannheim, Germany; 2 Department of Surgery, University Medical Center Mannheim, Medical Faculty Mannheim, University of Heidelberg, Theodor-Kutzer-Ufer 1-3, 68167 Mannheim, Germany; 3 Department of Internal Medicine II, University Medical Center Mannheim, Medical Faculty Mannheim, University of Heidelberg, Theodor-Kutzer-Ufer 1-3, 68167 Mannheim, Germany

**Keywords:** Endoscopic mucosal resection, Endoscopic submucosal dissection, Endoscopic full-thickness resection, Colorectal cancer, Early gastric cancer

## Abstract

**Objectives:**

The value of multidisciplinary tumor boards (MTBs) in the treatment of gastrointestinal cancer patients is well known. Most of the current evidence focuses on advanced cancer cases, whereas little is known about the effect of MTBs on early tumors, especially after endoscopic resection. The aim of our study is to evaluate the value of the MTB after endoscopic resection of malignant tumors of the gastrointestinal tract.

**Methods:**

We retrospectively analyzed all endoscopically resected malignant tumors in our department between 2011 and 2019, focusing on the existence of an MDT recommendation after endoscopic resection, the MDT adherence to the current guidelines, and the implementation of the recommendation by the patients.

**Results:**

We identified 198 patients fulfilling our inclusion criteria, of whom 168 (85%) were discussed in the MDT after endoscopic resection. In total, 155 of the recommendations (92%) were in accordance with the current guidelines, and 147 (88%) of them were implemented by the patients. The MDT discussion itself did not influence the overall survival, whereas the implementation of the MTB recommendation was associated with a significantly better prognosis. Deviations of the MDT recommendation from the guidelines had no effect on the overall survival.

**Conclusions:**

The discussion of endoscopically resected malignant tumors in the MTB is crucial for the treatment of patients with this type of cancer, since the implementation of the MTB recommendation, even if it deviates from the current guidelines, improves the prognosis.

A multidisciplinary tumor board (MTB) is a collaboration of specialists from a variety of different fields who gather to discuss oncological cases and make suggestions about further diagnostic and treatment options [1]. After their initial appearance in the 1920s, MTBs gained popularity in the late 1990s, and now they have become mandatory for the treatment of several malignancies in many countries, including the Netherlands, Germany, and the United Kingdom [2–4]. Gastrointestinal malignancies are among the most common entities presented in MTBs, both because of their high incidence and because of the complexity of the treatment [3, 5]. In most studies, the majority of the presented patients have advanced, metastatic, or recurrent cancer, whereas stage I tumors are less likely to be discussed [6]. The main reason for this patient selection is that surgical resection has traditionally been the first-line treatment for most early malignancies of the gastrointestinal tract, thus usually not requiring any input from other disciplines for the planning of the therapeutic strategy. However, the introduction of endoscopic resection as a curative treatment for low-risk early gastrointestinal malignancies has widened the therapeutic spectrum and revealed new challenges, including the correct indications for endoscopic resection and the evaluation of the necessity and planning of additional treatment after non-curative resections [4, 7–9]. Despite the increasing importance of the interdisciplinary management of these early malignancies, there are very few reports on MTB implementation before or after their endoscopic resection, focusing mainly on colorectal lesions [10, 11].

The aim of our study is to evaluate the implementation and outcomes of the MTB after endoscopic resection of malignant tumors of the gastrointestinal tract in a tertiary center.

## Materials and methods

We performed a thorough search through the endoscopic database of the Central Interdisciplinary Endoscopy Department of the University Medical Center of Mannheim and identified patients who had undergone endoscopic resection of histologically proven malignant tumors between 2011 and 2019. The periprocedural, histological, and follow-up data of the patients were extracted, pseudonymized, and retrospectively analyzed. The primary focus of the analysis was the presence of a documented MTB discussion, the adherence of the MTB to the current guidelines, and the implementation of the MTB recommendations by the patients, as well as the influence of those factors on the overall survival. A subgroup analysis according to the type and localization of the tumors was also performed when the number of patients was high enough for a statistical analysis.

The statistical analysis was performed with the use of IBM SPSS Statistics 28.0. Categorical variables were compared by a Chi-square test or Fischer’s exact test, and continuous variables were compared by Student’s *t* test or a Mann–Whitney test. The survival data were analyzed using Kaplan–Meier curves. Statistical significance was defined as *p* < 0.05. An approval of the ethics committee of the University of Heidelberg was acquired.

## Results

We identified 198 patients who underwent endoscopic resection of histologically proven malignant tumors in our department between 2011 and 2019; 128 patients were male and 70 female, with a mean age of 68 years (SD: 11.6 years). The resected tumors included squamous cell carcinomas and adenocarcinomas of the esophagus, gastric adenocarcinomas, adenocarcinomas of the colon and rectum, neuroendocrine tumors, ampullary carcinomas, and gastrointestinal stromal tumors (GISTs). The resection techniques included endoscopic mucosal resection (EMR), endoscopic submucosal dissection (ESD), and endoscopic full-thickness resection (EFTR). The exact distribution of the tumor entities and resection techniques is depicted in Table 1. The endoscopic resections were performed by eight experienced endoscopists with either a surgical or a gastroenterological background.

**Table 1:** Tumor entities, resection techniques and histological characteristics

	Patients (%)
Tumor entity	
Esophageal squamous cell carcinoma	6 (3%)
Esophageal adenocarcinoma	22 (11%)
Gastric adenocarcinoma	16 (8%)
Colonic adenocarcinoma	94 (48%)
Rectal adenocarcinoma	52 (26%)
Other	8 (4%)
Resection technique	
EMR	172 (87%)
ESD	24 (12%)
EFTR	2 (1%)
Macroscopic resection status	
Macroscopic complete resection	178 (90%)
Macroscopic incomplete resection	20 (10%)
T-stage	
Tis	47 (24%)
T1sm1	57 (29%)
T1sm2	10 (5%)
T1sm3	14 (7%)
T1smx**	56 (28%)
T2	14 (7%)
L-status	
L0	188 (95%)
L1	10 (5%)
V-status	
V0	194 (98%)
V1	4 (2%)
Grading	
G1	18 (9%)
G2	169 (85%)
G3	10 (5%)
G4	1 (1%)
R-status	
R0	98 (50%)
R1/Rx	80 (40%)
R2	10 (10%)
Curative resection	
Yes	40 (20%)
No	158 (80%)

Other: ampullary adenocarcinomas, neuroendocrine tumors, and gastrointestinal stromal tumors, T1smx  =  the exact depth of infiltration in the submucosal layer could not be accurately determined because of the fragmentation of the specimen

The macroscopic resection status was similar for all tumor entities. The distribution of the resection techniques was not even, with the majority of the colorectal tumors and the esophageal adenocarcinomas (Barrett carcinomas) being resected by EMR and with the ESD mainly used for esophageal squamous cell carcinomas and gastric adenocarcinomas (Table 2).

**Table 2:** Distribution of the resection techniques according to tumor entity

Tumor entity	EMR	ESD	EFTR	*p* < 0.0001
Esophageal squamous cell carcinoma (*n* = 6)	3 (50%)	3 (50%)	0	
Esophageal adenocarcinoma (*n* = 22)	22 (100%)	0	0	
Gastric adenocarcinoma (*n* = 16)	6 (37%)	10 (63%)	0	
Colonic adenocarcinoma (*n* = 94)	87 (93%)	5 (5%)	2 (2%)	
Rectal adenocarcinoma (*n* = 52)	45 (87%)	7 (13%)	0	

The histological characteristics of the tumors, including infiltration depth, lymphovascular and venous infiltration, grading, and resection status, are summarized in Table 1. The distribution of the histological characteristics was similar for all tumor entities.

In total, 168 (85%) patients were presented for discussion in the MTB after endoscopic resection (Group 1—MTB), and the remaining 30 patients received a recommendation regarding further treatment directly from the responsible physician (Group 2—no MTB). Although a few patients presented to our department with an already histologically diagnosed malignant tumor and were presented at the MTB both before and after the endoscopic resection, the majority of the cases were discussed only after the procedure and the recommendation was based on the final histology report of the specimen. The distribution of the tumor entities was similar in both groups. Tumor infiltration varied slightly, with more in situ carcinomas in Group 2 (Table 3), whereas the rest of the histological parameters were similar in both groups.

**Table 3:** Distribution of tumor infiltration between Group 1 and Group 2

T-stage	Group 1 (MTB, *n* = 168)	Group 2 (no MTB, *n* = 30)	*p*
Tis	33 (20%)	14 (47%)	0.046
T1sm1	51 (30%)	6 (20%)	
T1sm2	10 (6%)	0	
T1sm3	12 (7%)	2 (7%)	
T1smx	48 (29%)	8 (26%)	
T2	14 (7%)	0	

The MTB suggested follow-up in 69 cases (41%), further endoscopic resection in 10 cases (6%), transanal endoscopic microsurgery (TEM) in 8 cases (5%), oncologic surgery in 76 cases (45%), and chemotherapy/radiation therapy in 5 cases (3%). The MTB adherence to the German guidelines was 92% (155 patients), whereas 26 (87%) of the patients in Group 2 (no MTB) received a suggestion for further treatment according to the guidelines.

Furthermore, 147 patients (88%) complied with the MBT suggestion, including all patients with a suggestion for follow-up, whereas 21% of the patients with a suggestion for endoscopic resection, oncologic surgery, chemotherapy, or radiation refused any further treatment (Table 4).

**Table 4:** Compliance with the MTB suggestion

MTB suggestion	Complying patients	Noncomplying patients	*p*
Follow-up (*n* = 69)	69	0	< 0.0001
Endoscopic resection (*n* = 10)	8	2	
TEM (*n* = 8)	8	0	
Oncologic surgery (*n* = 76)	59	17	
Chemotherapy/radiotherapy (*n* = 5)	3	2	

**Fig. 1:**
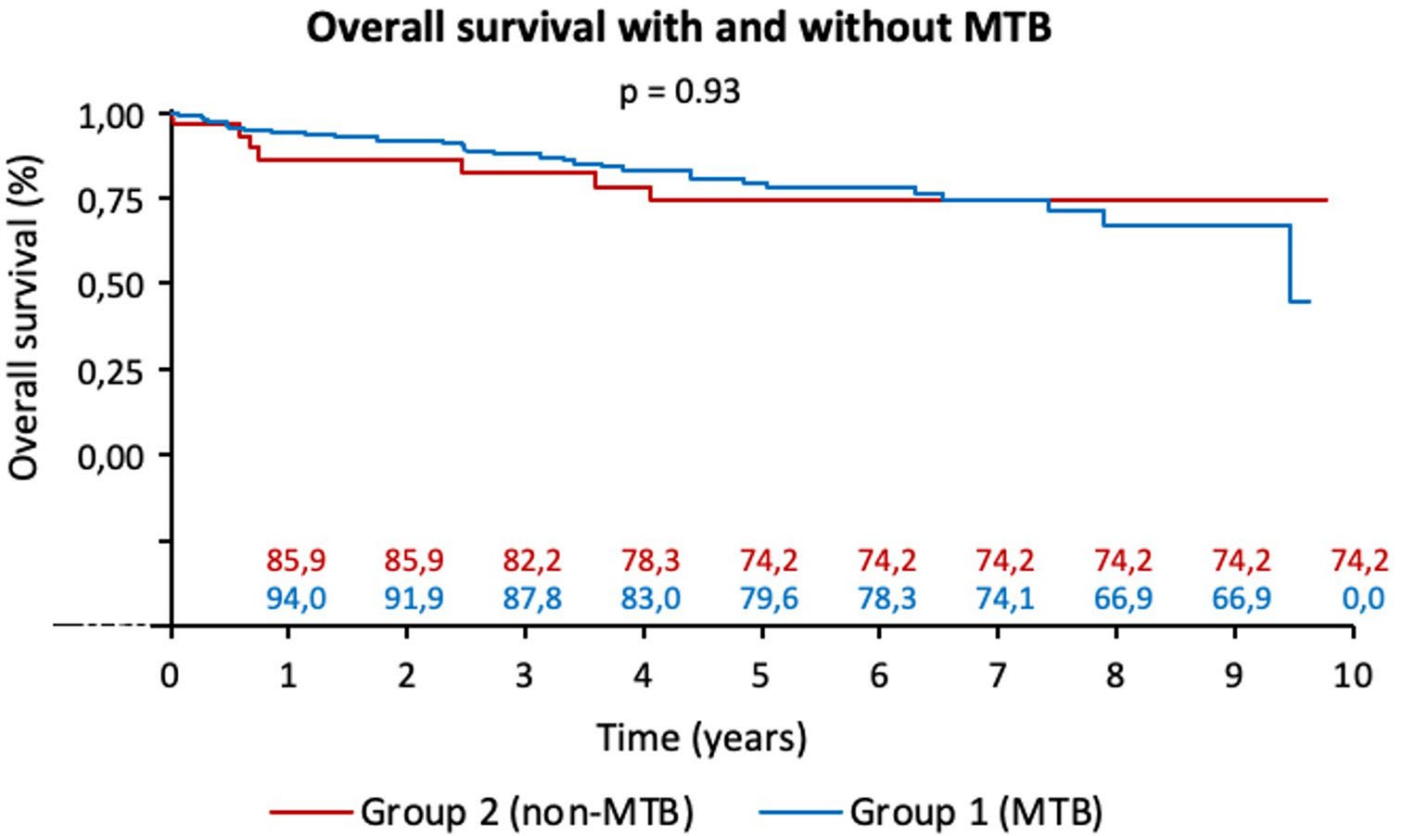
Overall survival with and without MTB

**Fig. 2:**
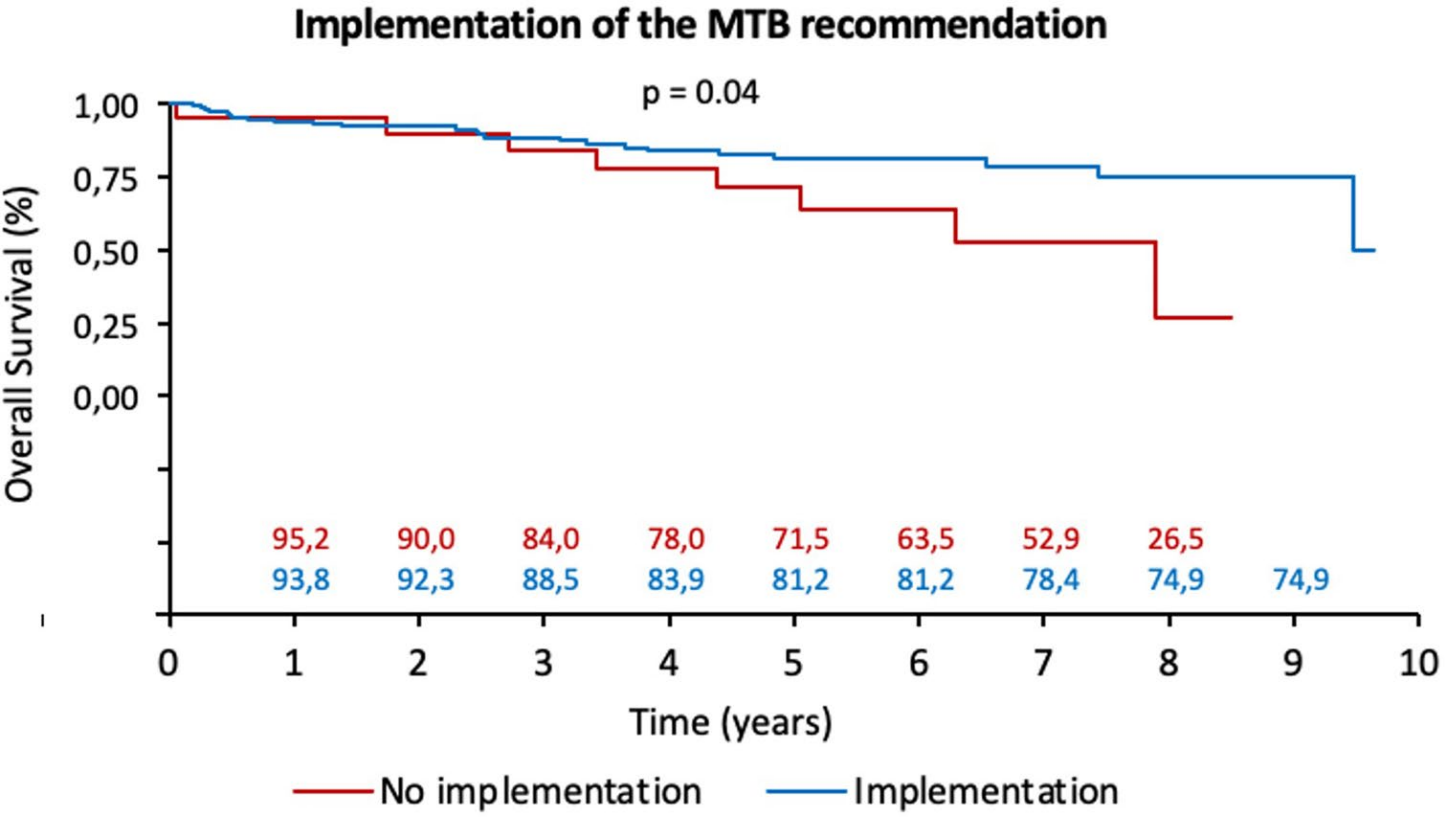
Implementation of the MTB recommendation

The median follow-up time was 43 months (range: 2–117 months). The discussion of the patients in an MTB did not influence the overall survival of the patients, which was similar in both groups (*p* = 0.93, Fig. 1). The adherence of the MTB to the guidelines did not affect the overall survival of the patients (*p* = 0.81), whereas for patients not discussed in the MTB, adherence to the guidelines was associated with a significantly better overall survival (*p* = 0.05). Among the patients in Group 1 (MTB), those who complied with the MTB suggestion had a significantly better overall survival (*p* = 0.04, Fig. 2).

## Discussion

The implementation of MTBs has been proven to be beneficial for many different types of cancer patients, especially for patients with gastrointestinal malignancies, so that nowadays they are an integral part of the oncologic management in most tertiary centers and even mandatory as part of the treatment guidelines in many countries [4, 12]. Most of the current literature focuses on patients with advanced disease, requiring a complex and often multimodal treatment, but there is hardly any evidence on MTBs for patients with stage I cancer, especially for those undergoing endoscopic resection [6, 13]. To our knowledge, this is the first study to assess the effects of an MTB discussion after endoscopic resection of malignant tumors of the gastrointestinal tract in a large cohort. Our data showed that the implementation of the MTB suggestions has a significant prognostic advantage for this group of patients. One of the main advantages is the possibility that in selected cases, the MTB team can deviate from the guidelines, taking into consideration the overall status of the patient and the current evidence, without compromising the quality of the treatment and the overall survival.

In the past, oncologic surgery was the only adequate therapeutic option for early gastrointestinal malignancies. Later studies have shown that, in a well-defined subgroup of early gastrointestinal cancers, the risk of lymphovascular invasion and positive lymph nodes is negligible, so that a complete local, organ-sparing resection of the tumor can be curative [14]. These data, in combination with the evolution of endoscopic resection techniques, led to endoscopic resection becoming the treatment of choice for early gastrointestinal tumors with a low risk of positive lymph nodes, a suggestion that has been integrated into the current guidelines [4, 7–9]. Nevertheless, the definition of local resectability is based on histological criteria, including depth of invasion, lymphovascular and venous invasion, and grading, which can be adequately assessed only in the resected specimen [14, 15]. Advanced endoscopic and imaging techniques allow us to predict local resectability to some extent, but their accuracy is still low, so that a multidisciplinary approach is crucial for setting the indication for endoscopic resection and especially for determining whether the procedure was curative, based on both periprocedural facts and the pathology report [16, 17]. A gastrointestinal MTB composed of endoscopists, surgeons, gastroenterologists, oncologists, radiologists, and pathologists is the perfect setting for this decision. As of 2010, we have therefore implemented the discussion of endoscopically resected malignant tumors in our MTB directly after endoscopic resection [18].

The cases in our cohort cover the entire spectrum of endoscopically resectable malignancies of the gastrointestinal tract, including epithelial lesions of the esophagus, the stomach, and the colorectum, gastrointestinal stromal tumors, and neuroendocrine tumors. The majority, though, were occult carcinomas in colorectal adenomas treated by piecemeal EMR (pmEMR). These cases pose a special challenge for the MTB, since a fragmented resection specimen rarely allows an accurate histopathological evaluation of the infiltration depth and the resection margins, so that the curative potential of the resection and the necessity for further treatment are difficult to assess [19, 20]. This explains the high number of T1smx and Rx tumors, and thus the overall low rate of complete and curative resections, in our study. ESD offers a higher en bloc resection rate and a better quality of the resection specimen, but its establishment in Europe was delayed in comparison to Asian countries [20, 21]. Therefore, in our center it was initially implemented to treat early gastric cancer and squamous cell carcinomas of the esophagus, whereas its use in the colorectum became more popular toward the end of the study period.

The selection of patients who need to be discussed in an MTB remains a controversial issue. The high caseload, especially in referral centers, as well as the involvement of multiple specialties in every MTB, is associated with high costs, while some authors argue that the discussion of many simple and straightforward cases per session leaves very little time to thoroughly discuss the more complex cases [22, 23]. Therefore, they suggest that only complex cases of advanced cancer should be discussed, while for low-risk cases recommendations should be made based on pre-agreed protocols [6, 24]. Nevertheless, we strongly believe that early gastrointestinal tumors should also be discussed on a multidisciplinary basis, in order to determine the curative potential of an endoscopic resection. In our cohort, different kinds of gastrointestinal malignancies were discussed in the MTB. In the first 2 years of our study period, the patient selection for the MTB relied on the discretion of the treating physician, whereas afterward the presentation of all cases became mandatory. This patient selection in the early phase of the study also explains the overrepresentation of in situ carcinomas in the non-MTB group.

Despite the long follow-up period and the large number of cases in our cohort, we were not able to find a survival benefit for the patients of the MTB group. This might partially be due to the smaller size and the previously mentioned overrepresentation of earlier tumor stages in the non-MTB group, but it also agrees with the findings of several large multicenter studies and meta-analyses [12, 25]. A few studies showed a positive effect of MTBs on survival, but they mostly compared large cohorts before and after MTB implementation and therefore patients treated in different time frames, potentially depicting the development in treatment options and the overall improvement in the prognosis of these diseases through time [26, 27]. On the other hand, a prospective, randomized comparison, which could eliminate both the difference in time frame and the selection bias, is at the moment ethically unacceptable because of all the other proven benefits of MTBs. This is probably the reason why many studies on MTBs do not discuss the issue of survival, and the evidence on their prognostic relevance is low [18]. Nevertheless, even if a direct correlation between MTBs and prognosis is currently not possible, MTBs play an important role in the standardized algorithms implemented in certified cancer centers and have been associated with a significant improvement in overall survival [28].

A further argument often raised against MTBs is the existence of detailed guidelines for most of the discussed diseases. However, one of the main roles of an MTB is not just to implement the guidelines but also to offer an individualized treatment strategy for every patient, taking into account the patient’s clinical condition, comorbidities, and wishes [18]. In our study, MTB adherence to the guidelines was 92%, similar to the findings of other studies [1, 29]. Brauer et al. reported an adherence of 100% in 470 cases of pancreatic and upper gastrointestinal cancer, albeit with an implementation rate of only 70%, thus raising the issue of how realistic the recommendations of the guidelines can be in certain cases [30]. Apart from that, the development of guidelines, particularly for malignant diseases, is a long process, so that new developments need several years until they are integrated into the guidelines. Especially in the case of early gastrointestinal tumors, large studies of the past few years have expanded the criteria of local resectability for both gastric and colorectal cancer, thus reducing the necessity of oncologic surgery, but these recent developments have not been integrated into the guidelines yet [15, 31]. Therefore, small deviations from the guidelines, based either on the patients’ status or on the current evidence, are acceptable, as long as they are well documented and discussed in a multidisciplinary setting. Our data confirm this argument by showing that in selected cases, MTB deviation from the guidelines has no negative effect on the prognosis, whereas in the non-MTB group this deviation was associated with significantly worse survival.

One of the most important aspects of recommendations, especially for cancer patients, is the degree of implementation. The multidisciplinary discussion itself makes patients feel that they are being taken seriously, thus increasing patient satisfaction and readiness to follow the physician’s recommendations [32]. The implementation rate of MTB recommendations varies greatly among different studies, ranging from 60 to 100% [33, 34]. More aggressive treatment recommendations, including radical surgery and chemotherapy, are less likely to be implemented, a fact also shown in our study [35]. The main reasons for poor adherence to the MTB recommendations are patients’ comorbidities and wishes and rarely new information that was acquired after the MTB discussion [34]. Taking those factors into consideration during the MTB discussion is therefore crucial and could potentially increase the implementation rate. The importance of the patients’ adherence to the MTB recommendations is clearly shown in our study, since it was associated with a significant survival benefit, regardless of the compliance with the guidelines.

To our knowledge, our study is the first to address the effects of MTBs after endoscopic resection of gastrointestinal malignancies. The study’s main strengths are the large number of patients included and the long follow-up period. Additionally, both MTB and non-MTB patients were treated during the same time frame, thus reducing the influence of improvements in treatment options during the study period. On the other hand, the retrospective design of the study and the smaller size of the control group are the main weaknesses. However, nowadays most centers have integrated MTB discussions into their treatment algorithms, so that the number of patients not discussed in an MTB will always be smaller. A prospective, randomized trial would currently be very difficult to plan and execute and would probably also be unethical, for the reasons explained earlier. Therefore, we believe that the design of our study addresses this subject in the best possible way.

Based on our findings, we can conclude that an MTB discussion after endoscopic resection of early gastrointestinal malignant tumors is crucial for the planning of further treatment. The MTB should take patients’ comorbidities and wishes, as well as new scientific evidence, into consideration, and in selected cases the MTB could deviate from the current guidelines, without compromising the prognosis. Special attention should be given to the correct implementation of the MTB recommendations, since this is a crucial factor for the improvement of the overall survival.
